# Black Americans’ Diminished Health Returns of Professional Occupations: A Thirty-Year Follow-Up Study of Middle-Aged and Older Adults

**DOI:** 10.1007/s40615-024-02034-9

**Published:** 2024-05-28

**Authors:** Shervin Assari

**Affiliations:** 1 Department of Internal Medicine, Charles R Drew University of Medicine and Science, Los Angeles, CA USA; 2 Department of Urban Public Health, Charles R Drew University of Medicine and Science, Los Angeles, CA USA; 3 Department of Family Medicine, Charles R Drew University of Medicine and Science, Los Angeles, CA USA; 4 Marginalization-Related Diminished Returns Center, Los Angeles, CA USA

**Keywords:** Ethnic groups, Occupation, Employment, Socioeconomic status

## Abstract

**Introduction:**

Occupational classes exert substantial effects on both subjective and objective health outcomes. However, it remains unclear whether the health impact of similar occupational classes varies across racial groups among middle-aged and older adults in the United States.

**Aim:**

Grounded in the theory of Minorities’ Diminished Returns (MDRs), which posits that health benefits from resources such as employment are systematically weaker for racial minority populations, particularly Non-Latino Black individuals, this study tested Black-White disparities in the effects of similar occupational classes on health outcomes in middle-aged and older adults.

**Methods:**

Utilizing data from the Health and Retirement Study (HRS), we employed a 30-year longitudinal design with a nationally representative sample of middle-aged and older adults in the United States. Six occupational classes—operator, managerial, professional specialty, sales, clerical/admin, and service—served as the key predictor variables (independent variables), with race as the moderator. Various health outcomes, including self-rated health, chronic disease, body mass index (BMI), activities of daily living (ADL), and cognitive function, were measured longitudinally from wave 1 to wave 15 (from baseline to 30 years later). Statistical analyses, incorporating logistic regression models, were conducted to assess associations between occupational class and health outcomes overall and based on race.

**Results:**

Our analysis included 7538 Non-Latino White or Non-Latino Black participants followed for up to 30 years. Initial findings revealed positive health effects of professional occupations on cognitive function and self-rated health over 30 years of follow-uWe also identified significant interactions between race and professional occupational class on all health outcomes, indicating notable racial differences in the effects of professional occupations on health outcomes across domains. The effects of professional occupational class were weaker for Non-Latino Black individuals than for Non-Latino White individuals.

**Conclusion:**

Consistent with the Minorities’ Diminished Returns theory, our findings indicated less pronounced positive effects of professional occupations on a wide range of health outcomes for Non-Latino Black individuals compared to Non-Latino Whites. These disparities emphasize the critical need to address structural factors that contribute to the diminished returns of prestigious occupations for Non-Latino Black populations.

## Introduction

Occupational classes play a pivotal role in shaping various aspects of population and individual health, intricately linking educational achievements to health outcomes through work and employment [1]. Beyond their conventional significance, the nature of one’s occupation significantly influences various dimensions of well-being [2]. Manual jobs often entail high stress and demand, while white-collar occupations tend to be less stressful [3].

Due to the pervasive influence of racism, social stratification, and segregation, the relationship between work, occupation class, and health becomes complex, non-linear, and non-additive, particularly when viewed through the lens of race [4]. Historical legacies such as Jim Crow and segregation have contributed to divergent occupational trajectories for Non-Latino Black and Non-Latino White individuals, influencing their choices and opportunities [5]. Understanding the disparities in health across similar occupations for Non-Latino Black and Non-Latino White people necessitates recognizing the lingering effects of systemic racism, labor market discrimination, and historical segregation [6]. This nuanced knowledge is essential for unraveling the intricate interplay between education, occupation, and health outcomes specific to race [7].

Recent research on Minorities’ Diminished Returns (MDRs) [8] has shed light on the nuanced effects of educational attainment on health, happiness, and illness prevention, consistently revealing weaker outcomes for Non-Latino Black individuals compared to their Non-Latino White counterparts [9–13]. While educational disparities and their impact on health have been explored [14–17], the extension of these diminished returns to occupational classes remains a less-charted territory, particularly among middle-aged and older adults in the United States [18–20].

Some of the occupational classes such as managerial, professional/specialty, sales, and clerical admin are associated with lower stress and manual labor and may have lower tear and wear [21]. In contrast, occupations in the areas of service and operator roles exhibit noticeable difficulty [22]. There are at the same time more Non-Latino Black individuals in the occupations such as service and operator, and there are more managerial, professional, and clerical/admin position for Non-Latino White individuals [23]. This labor market division is in part due to lower education of Non-Latino Black people; however, a large proportion of such division is due to lower quality of education and labor market discrimination and segregation that direct Black people to service and manual labor and Non-Latino White people to more prestigious lower stress higher job pays [24]. As a result, there are fewer representation of Black people with same education in managerial, professional/specialty, and clerical admin positions, with a higher prevalence in service and operator roles, again, across same levels of education [25]. This racial-based occupational stratification carries broader health implications, as the nature of work in these categories can contribute to differential stressors and wear-and-tear on the body [26].

Systemic barriers include discriminatory hiring practices and limited access to career advancement opportunities [27]. These barriers contribute to the overrepresentation of Non-Latino Black individuals in service and operator positions, often involving manual labor and associated with higher levels of physical and psychological stress [28].

Despite the underrepresentation of Non-Latino Black individuals in professional/specialty and managerial roles [29], there is a remaining question that if Non-Latino Black and Non-Latino White individuals end up in such low stress higher pay jobs, would we still see diminished health returns of occupational attainment or not for Non-Latino Black than Non-Latino White people [18–20].

Manual jobs, such as those in service and operator categories, are characterized by elevated stressors and wear-and-tear due to the physical demands of the work [30, 31]. This differential recruitment to various occupational classes, driven by systemic factors, may be a contributing factor to observed health disparities by race [32]. The overrepresentation of Black individuals in manual jobs may expose them to heightened health risks, potentially explaining part of the health disparities seen across racial lines. Recognizing and addressing these occupational disparities is crucial for advancing health equity, ensuring fair access to opportunities across job classes, and fostering environments where individuals of all races can thrive [33].

Labor market discrimination persists as a challenge [34–36], leading to stark occupational disparities between Non-Latino Black and Non-Latino White individuals even when educational qualifications are comparable [37]. Deep-seated biases within hiring processes and workplace structures result in Non-Latino Black individuals facing barriers that funnel them into lower-status, less remunerative occupations [38]. This discrimination occurs at various stages, from hiring decisions to promotions, perpetuating racial wage gaps and socioeconomic inequalities [39–41]. Beyond economic disparities, occupational segregation reinforces social stratification, limiting access to resources and opportunities for career progression. Addressing labor market discrimination is crucial for dismantling these occupational disparities and fostering a more equitable and inclusive workforce based on merit rather than discriminatory practices [42, 43].

This study seeks to contribute significantly to existing literature by exploring the intricate associations between occupational classes, race, and an extensive spectrum of health outcomes in middle-aged and older adults. By expanding the MDRs framework beyond educational attainment to occupational classes, the investigation aims to offer a comprehensive understanding of how historical and contemporary societal structures perpetuate health disparities. Leveraging extensive longitudinal data from the Health and Retirement Study (HRS) [44–48], the study will shed light on whether occupational classes differentially influence the health trajectories of Non-Latino Black and Non-Latino White individuals, addressing a critical gap in the understanding of MDRs [49–53] within the context of aging populations. As disparities in occupational attainment persist [54], uncovering the health implications is imperative for developing targeted interventions and policies that address the unique needs of minority populations, advancing efforts toward health equity, and fostering the well-being of middle-aged and older adults across diverse demographic backgrounds in the United States.

## Aims

In this study, we aim to examining the additive and multiplicative effects of occupational classes [55–60] on various dimensions of health among a nationally representative sample of middle-aged and older adults in the United States. Utilizing data from the Health and Retirement Study (HRS) [44–48] with up to 30 years of follow-up, our goal is to illuminate the specific ways in which educational attainment interacts with race, particularly among Non-Latino Black populations, to shape health outcomes in the later stages of life. The results of this investigation aim to contribute to a deeper understanding of the complex interplay between occupational classes, race, and various aspects of health. Ultimately, these insights may inform targeted interventions and policies designed to enhance well-being across diverse demographic groups of middle-aged and older adults in the United States.

## Methods

### Design and Setting

Data were obtained from all available biennial waves of the HRS [45] for 1992 to 2020. The HRS collects extensive data on various aspects of participants, including demographic, socioeconomic, social, psychological, economic, employment, and health data, as well as health behaviors and health service utilization. HRS data has also measured a wide range of data related to retirement including time of retirement [47]. Data were collected through telephone or face-to-face interviews, and proxy interviews were used for participants who were unavailable. Detailed information on the study design and methodology can be found elsewhere [44–48]. We used the RAND HRS Longitudinal File [61] that was publicly released in March 2023.

### Sample and Sampling

The HRS features a cohort-based longitudinal design, with the first cohort recruited in 1992. The HRS used a national area probability sample to recruit participants aged 51 to 61 at baseline in 1992. For the current analysis, only the core (primary) sample recruited in 1992 was included to offer the longest follow-up period. All our HRS participants were born between 1931 and 1941, and the sample reflects all middle-aged and older adults aged 51–61 residing in US households in 1992 (baseline = wave 1).

### Analytical Sample

The analytical sample for this study included US adults aged 51–61 in 1992 who identified as Non-Latino White or Non-Latino Black. Individuals from other racial groups were not included in the analysis. All participants from the HRS core sample were eligible for analysis regardless of the duration of follow-up or time of mortality, except for those who identified as retired at baseline. The analytical sample consisted of 7538 non-retired working participants at baseline who were followed for up to 30 years and were either Non-Latino White or Non-Latino Black.

### Measures

#### Outcomes

This study included five health outcomes namely SRH, chronic disease, BMI, cognitive function, and disability. These variables were all binary (0 = good and 1 = poor). These measures were defined based on biannual measures. Although this variable was continuous, we used k-mean cluster analysis to define risk categories over the 30 years of follow-uAll our outcomes were always poor condition of SRH, chronic disease, BMI, cognitive function, and disability.

#### Self-Rated Health

Using the conventional measure of self-rated health, HRS has asked participants to rate their health. Research has shown that poor SRH predicts mortality, net of other independent risk factors. SRH was measured every 2 years from 1992 to 2016. Respondents reported their overall health on a five-point scale as excellent (1), very good (2), fair (3), good (4), and poor (5) [62–64]. We treated SRH as an ordinal variable, ranging from 1 to 5, with a higher score indicating worse self-rated health.

#### Chronic Conditions

Participants reported whether they had conditions such as heart disease, arthritis, asthma, diabetes, and depression. Each condition was coded 1 if present and coded as 0 if absent.

#### Body Mass Index

Height and weight were measured in wave 10. BMI was calculated as the person’s weight in kilograms divided by the square of height in meters. BMI was operationalized as a continuous measure. Although this variable was continuous, we used k-mean cluster analysis to define risk categories over the 30-year follow-up period.

#### Instrumental Activities of Daily Living

Respondents were asked if they need assistance for activities such as using bathroom, changing clothes, and grocery shopping. Each item was a 0–1 item with higher score indicating higher disability (physical dysfunction).

#### Cognitive Function

Cognitive function was measured using TICS. Introduced in 1988 by Brandt and colleagues, TICS is one of most extensively used measures in field surveys of cognitive functioning [65].

### Predictor

#### Occupation classes

Using Census 1980, the HRS has generated 17 occupational classes that are as follows: 01. Managerial specialty operators, 02. Prof specialty opr/tech support, 03. Sales, 04. Clerical/admin supp, 05. Svc:prv hhld/clean/bldg svc, 06. Svc:protection, 07. Svc:food prep, 08. Health services, 09. Personal services, 10. Farming/forestry/fishing, 11. Mechanics/repair, 12. Constr trade/extractors, 13. Precision production, 14. Operators: machine, 15. Operators: transport, etc., 16. Operators: handlers, etc., and 17. Member of Armed Forces. We reduced these classes to the six following groups: 1. Managerial and specialty operations, 2. Professional Specialty, 3. Sales, 4. Clerical/admin supp, 5. Services, and 6. Manual. Due to low sample size, we did not include 6 HRS participants whose occupational class was dropped [66]. This variable was measured at baseline in 1992.

### Controls

#### Educational attainment

Educational attainment was measured as the following five categories: This variable was treated as a categorical variable with less than high school as the reference category. Educational attainment was self-reported at baseline in 1992.

#### Age

Age (years) was treated as a continuous variable, calculated based on the number of years since birth.

#### Gender

Gender was treated as a dichotomous variable (coded as 0 for female and 1 for male).

#### Family Structure

Participants reported if they were married at each wave. We used a dichotomous variable for married (coded as 1) and any other status (coded as 0).

### Data Analysis

Data were analyzed using SPSS 25.0 (IBM Corporation, Armonk, NY, USA). Univariate analyses included reporting means (standard deviation [SD]) and absolute/relative frequencies (*n* and %). Multivariable models included two sets of logistic regression models for each outcome. Both regression models were run in the pooled sample (analytical sample): model 1 without interaction and model 2 with the statistical interaction terms between race and occupational classes. Model 2 included six interaction terms as follows: race × managerial, race × professional specialty, race × sales, race × clerical admin, and race × service. In all our models, occupational classes were the predictor variable, a health outcome over up to 30 years (categorical variable) was the outcome, and race was the moderator, while controlling for educational attainment, gender and age were confounders. Models were tested with and without interaction terms to assess the significance of racial differences in the relationships between educational attainment and life satisfaction. Before running the models, multicollinearity between study variables was checked. Odds ratio, 95% confidence interval, and *p* values were reported.

### Ethics Statement

The HRS study protocol was approved by the University of Michigan Institutional Review Board. All HRS participants signed written consent. The data were collected, stored, managed, and analyzed in a fully anonymous fashion. As we used fully de-identified publicly available data, this study was non-human subject research, according to the NIH definition.

## Results

Overall, 7538 entered our analysis and were followed for up to 30 years. Table 1 presents descriptive data overall and by race. From the total participants, 17% were Non-Latino Black. At baseline, 15.5%, 16.2%, 10.1%, 16.5%, 14.8%, and 26.8% were in managerial, professional, sales, clerical admin, service, and operator occupations. The average age at baseline was 54.53 (SD = 5.17). From all participants, 32.1%, 17.7%, 12.8%, 7.2%, and 15.6% had poor health in the domains of SRH, chronic conditions, disability, BMI, and cognitive function over the 30-year follow-up period. Non-Latino Black participants were more likely than Non-Latino White participants to have poor SRH, chronic conditions, disability, BMI, and cognitive function over the 30-year follow-up period.

**Table 1:** Descriptive data overall and by race

	All		Non-Latino White		Non-Latino Black	
	*n*	%	*n*	%	*n*	%
Race						
Non-Latino White	6255	83.0	6255	100	0	0
Non-Latino Black	1283	17.0	0	0	1283	100
Sex*						
Female	3793	50.3	3040	48.6	753	58.7
Male	3745	49.7	3215	51.4	530	41.3
Education*						
Lt high school	1376	18.3	918	14.7	458	35.7
GED	387	5.1	335	5.4	52	4.1
High school graduate	2589	34.3	2216	35.4	373	29.1
Some college	1603	21.3	1370	21.9	233	18.2
College and above	1583	21.0	1416	22.6	167	13.0
Occupation*						
Managerial*	1169	15.5	1073	17.2	96	7.5
Professional*	1221	16.2	1060	16.9	161	12.5
Sales*	765	10.1	710	11.4	55	4.3
Clerical admin*	1246	16.5	1093	17.5	153	11.9
Service*	1113	14.8	715	11.4	398	31.0
Operator*	2017	26.8	1597	25.5	420	32.7
Missing	7	0.1	7	.1		
Self-rated health*						
Good	5121	67.9	4442	71.0	679	52.9
Poor	2417	32.1	1813	29.0	604	47.1
Chronic medical conditions*						
Good	6206	82.3	5187	82.9	1019	79.4
Poor	1332	17.7	1068	17.1	264	20.6
Disability*						
Good	6003	79.6	5786	92.5	966	81.9
Poor	962	12.8	469	7.5	213	18.1
Body mass index (BMI) *						
Good	6997	92.8	5862	93.7	1135	88.5
Poor	541	7.2	393	6.3	148	11.5
Cognitive dysfunction						
Good	5522	73.3	4857	77.6	665	51.8
Poor	1174	15.6	708	11.3	466	36.3
Missing	842	11.2	690	11.0	1131	88.2
	Mean	SD	Mean	SD	Mean	SD
Baseline age	54.53	5.17	54.58	5.19	54.28	5.11

*p < 0.05

As shown in Table 2, those in professional occupations had lower odds of being in poor cognitive and SRH classes over the 30-year follow-up period.

**Table 2:** Results of model 1 without statistical interactions between race and occupational classes on various aspects of health

	Self-rated health (SRH)				High chronic medical conditions (CMC)				High disability				High body mass index (BMI)				Poor cognitive function			
	OR	95%	CI	*p*	OR	95%	CI	*p*	OR	95%	CI	*p*	OR	95%	CI	*p*	OR	95%	CI	*p*
Male	1.13	1.00	1.28	0.044	0.96	0.83	1.10	0.532	1.03	0.88	1.21	0.718	0.70	0.56	0.86	0.001	1.55	1.30	1.84	<0.001
Age (baseline)	1.03	1.02	1.04	<0.001	1.06	1.05	1.08	<0.001	1.06	1.05	1.08	<0.001	0.96	0.94	0.98	<0.001	1.09	1.07	1.11	<0.001
Non-Latino Black	1.71	1.50	1.96	<0.001	1.06	0.90	1.24	0.510	1.31	1.10	1.57	0.003	1.72	1.39	2.13	<0.001	3.86	3.26	4.57	<0.001
Education				<0.001				<0.001				0.003				0.406				<0.001
Less than high school	Ref																			
GED	3.65	2.98	4.46	<0.001	1.98	1.56	2.51	<0.001	1.67	1.27	2.19	<0.001	1.27	0.89	1.83	0.193	10.27	7.37	14.30	<0.001
High school graduate	2.63	2.02	3.42	<0.001	1.90	1.39	2.58	<0.001	1.57	1.10	2.25	0.013	1.54	0.97	2.43	0.066	4.17	2.78	6.26	<0.001
Some college	1.79	1.50	2.15	<0.001	1.38	1.12	1.72	0.003	1.25	0.98	1.60	0.077	1.30	0.95	1.78	0.107	2.69	1.95	3.72	<0.001
College and above	1.53	1.27	1.84	<0.001	1.41	1.14	1.75	0.002	1.28	1.00	1.64	0.053	1.17	0.85	1.61	0.323	1.92	1.38	2.68	<0.001
Occupation																				
Operator	Ref																			
Managerial	0.67	0.56	0.80	<0.001	0.87	0.70	1.08	0.207	0.74	0.57	0.96	0.021	0.93	0.66	1.29	0.653	0.40	0.30	0.53	<0.001
Professional specialty	0.71	0.58	0.88	<0.001	0.89	0.69	1.13	0.332	0.80	0.61	1.07	0.130	0.97	0.68	1.40	0.878	0.51	0.37	0.71	<0.001
Sales	0.84	0.69	1.02	0.078	0.91	0.72	1.15	0.436	1.08	0.84	1.40	0.540	0.94	0.66	1.35	0.752	0.58	0.43	0.76	<0.001
Clerical admin	0.86	0.72	1.03	0.104	0.92	0.74	1.15	0.478	0.93	0.73	1.19	0.574	0.94	0.69	1.29	0.710	0.44	0.33	0.58	<0.001
Service	1.14	0.96	1.34	0.126	1.32	1.09	1.60	0.004	1.18	0.95	1.47	0.143	1.25	0.94	1.66	0.120	1.01	0.82	1.25	0.917
Constant	0.05			<0.001	0.01			<0.001	0.00			<0.001	0.63			0.341	0.00			<0.001

As shown in Table 3, there were statistically significant interactions between professional occupations and race on the odds of being in poor health across all outcomes over the 30-year follow-up period. These interactions suggested that the protective effects of professional occupations on the odds of being in poor health were weaker for Non-Latino Black individuals compared to Non-Latino White individuals.

**Table 3:** Results of model 2 with statistical interactions between race and occupational classes on various aspects of health

	SRH				CMC				Disability				BMI				Cog			
	OR	95%	CI	*p*	OR	95%	CI	*p*	OR	95%	CI	*p*	OR	95%	CI	*p*	OR	95%	CI	*p*
Male	1.13	1.00	1.28	0.042	0.97	0.84	1.11	0.639	1.03	0.88	1.22	0.700	0.70	0.57	0.87	0.001	1.55	1.30	1.85	<0.001
Age (baseline)	1.03	1.02	1.04	<0.001	1.06	1.05	1.08	<0.001	1.06	1.05	1.08	<0.001	0.96	0.94	0.97	<0.001	1.09	1.07	1.11	<0.001
Non-Latino Black	1.48	1.19	1.85	0.001	0.96	0.73	1.26	0.762	1.05	0.77	1.43	0.768	1.25	0.83	1.88	0.294	3.02	2.32	3.93	<0.001
Education				<0.001				<0.001				0.002				0.418				<0.001
Less than high school	Ref																			
GED	3.68	3.00	4.51	<0.001	1.99	1.57	2.53	<0.001	1.70	1.29	2.23	<0.001	1.30	0.91	1.87	0.155	10.33	7.41	14.40	<0.001
High school graduate	2.61	2.01	3.40	<0.001	1.91	1.40	2.60	<0.001	1.55	1.08	2.21	0.017	1.53	0.96	2.41	0.071	4.12	2.75	6.20	<0.001
Some college	1.79	1.50	2.15	<0.001	1.38	1.11	1.71	0.003	1.24	0.97	1.59	0.088	1.29	0.94	1.78	0.112	2.70	1.95	3.73	<0.001
College and above	1.53	1.27	1.84	<0.001	1.41	1.14	1.76	0.002	1.26	0.99	1.62	0.066	1.17	0.85	1.61	0.322	1.93	1.38	2.70	<0.001
Occupation																				
Operator	Ref																			
Managerial	0.66	0.55	0.80	<0.001	0.85	0.67	1.06	0.150	0.68	0.52	0.90	0.006	0.82	0.57	1.18	0.287	0.40	0.29	0.54	<0.001
Professional specialty	0.65	0.52	0.81	<0.001	0.79	0.61	1.03	0.076	0.71	0.53	0.97	0.030	0.77	0.51	1.16	0.208	0.42	0.29	0.62	<0.001
Sales	0.82	0.67	1.00	0.049	0.91	0.72	1.16	0.462	1.07	0.82	1.40	0.617	0.90	0.61	1.32	0.587	0.54	0.40	0.74	<0.001
Clerical admin	0.81	0.67	0.99	0.036	0.97	0.77	1.22	0.774	0.84	0.64	1.10	0.207	0.90	0.64	1.26	0.537	0.41	0.29	0.56	<0.001
Service	1.12	0.92	1.36	0.256	1.29	1.03	1.62	0.028	1.16	0.89	1.51	0.260	1.12	0.79	1.59	0.527	0.84	0.65	1.10	0.208
Race × occupation																				
Race × managerial	0.93	0.55	1.58	0.788	1.26	0.67	2.37	0.475	1.74	0.87	3.45	0.115	1.80	0.81	4.00	0.151	0.87	0.41	1.87	0.727
Race × professional specialty	1.68	1.09	2.58	0.018	2.06	1.24	3.42	0.005	1.90	1.07	3.39	0.030	2.56	1.32	4.95	0.005	1.95	1.06	3.58	0.032
Race × sales	1.18	0.64	2.19	0.597	0.87	0.38	2.00	0.744	0.76	0.30	1.96	0.577	0.97	0.31	3.04	0.957	1.24	0.56	2.75	0.599
Race × clerical admin	1.41	0.93	2.15	0.103	0.66	0.36	1.20	0.173	1.81	1.04	3.15	0.035	1.15	0.57	2.33	0.687	1.37	0.77	2.42	0.280
Race × service	1.11	0.79	1.56	0.538	1.13	0.76	1.68	0.537	1.15	0.73	1.79	0.549	1.46	0.83	2.55	0.187	1.66	1.11	2.50	0.014
Constant	0.05			0.000	0.01			<0.001	0.00			<0.001	0.71			0.472	0.00			<0.001

## Discussion

Leveraging the 30-year follow-up data from the comprehensive Health and Retirement Study, encompassing a nationally representative sample of Non-Latino Black and Non-Latino White individuals, our investigation unveiled two noteworthy observations. Firstly, positive associations were observed between occupational classes such as professional and managerial and a diverse array of objective and subjective mental and physical health outcomes. Secondly, our findings exposed Black-White disparities in the impact of professional occupational class on health outcomes among middle-aged and older adults in the United States, with a disadvantageous impact on Non-Latino Black individuals.

Our initial result aligns with existing literature consistently demonstrating a positive correlation between occupational classes like managerial and professional and various health outcomes [1, 67, 68]. This correlation is attributed to multiple mechanisms, including lower levels of wear and tear, reduced exposure to stressors, and higher remuneration [69–72]. Professions within these occupational classes typically offer superior benefits, such as improved healthcare accessibility, better working conditions, and contribute to the maintenance of individual health trajectories [73]. Occupations associated with higher status and prestige are linked to numerous favorable health outcomes due to increased resource access and diminished exposure to adverse working conditions [74].

Our second finding is consistent with recent research on Minorities’ Diminished Returns [8], a theory positing that the economic and health advantages derived from resources like education, employment, and income are systematically weaker for racial minority populations, particularly Non-Latino Blacks with a history of slavery, compared to the most privileged social group (Non-Latino Whites). This theory illuminates the persistence of racial health disparities, influencing individuals’ experiences and outcomes even when educational and occupational achievements are comparable. It underscores the inadequacy of socioeconomic status alone in explaining these disparities, emphasizing the necessity for comprehensive interventions to address and close racial gaps visible across all socioeconomic status levels. This concept suggests that the positive effects of educational attainment on health, happiness, and preventive outcomes on illness and depression are less pronounced for Non-Latino Black individuals compared to their Non-Latino White counterparts [75, 76]. Attributed to racism, social stratification, and segregation, Minorities’ Diminished Returns theory posits that education’s contribution is most substantial for those not facing racialization, while for individuals encountering discrimination and blocked opportunities with lower quality education, educational attainment may generate fewer positive outcomes [9, 77].

Before, Minorities’ Diminished Returns theory was never tested with occupational classes of middle-aged and older adults [78–80]. Thus, this was the first study that investigated whether Minorities’ Diminished Returns phenomenon can be seen for the effects of occupational classes on health of Non-Latino Black middle age and older people in the United States [81].

The historical legacy of slavery, coupled with the ongoing persistence of racism manifested through segregation and social stratification, remains a potent force shaping the experiences of racial minorities today [82]. This historical and contemporary backdrop significantly influences the access that racial minorities have to resources and opportunities [83]. The lasting effects of both historical and current inequalities compound the disparities in the benefits derived from occupational class, impeding the advancement of racialized groups [84]. Systemic factors, including discrimination, barriers created by structural racism, and constrained access to high-quality occupations, collectively contribute to the reduced returns of occupational class for minorities [85]. Recognizing and addressing these deep-rooted challenges is essential for dismantling barriers and fostering a more equitable landscape for all [86–88].

### Implications

The implications of our findings extend to the realms of research, policy, and practice, urging targeted initiatives to address disparities in the impact of occupational class on health outcomes. Efforts should be directed toward developing tailored policies, programs, and interventions specifically designed to dismantle structural and institutional racism, focusing on the removal of barriers hindering the professional advancement of Non-Latino Black individuals. This includes addressing challenges unique to professional Non-Latino Black people. Improving healthcare accessibility and ensuring that the benefits of professional positions reach Non-Latino Black individuals are crucial components of an inclusive strategy. Moreover, combating discriminatory practices within the labor market and educational systems is paramount, with policies crafted to account for the diminished impacts of high-prestige occupations, such as those within the professional class, on Non-Latino Black populations. It is imperative that endeavors to enhance occupational outcomes translate into tangible improvements in health outcomes for all, fostering a more equitable and inclusive society.

### Limitations

While our study provides valuable insights, it is not without limitations. One notable constraint is the reliance on self-reported health measures, including chronic diseases and overall health. It is essential to acknowledge that these self-reported instruments may exhibit differential psychometric properties based on race, introducing a potential source of bias. Furthermore, the dataset’s exclusive focus on middle-aged and older adults may limit the generalizability of our findings across the entire lifespan. The presence of unmeasured confounders is another aspect that warrants consideration, as their influence could impact the study’s outcomes. Additionally, it is important to note that our study solely incorporated individual-level data, lacking the inclusion of area-level variables. This omission limits our ability to explore the potential impact of broader contextual factors on the observed health patterns. Future research endeavors should aim to address these limitations for a more comprehensive understanding of the complex interplay between health outcomes and various influencing factors.

### Future Directions of Research

Future research endeavors should delve into the intricacies within race and ethnicity to gain a more nuanced understanding of the diverse experiences that contribute to observed health patterns. Qualitative studies, with their ability to capture real-life experiences, are essential in unraveling the complexities underlying these patterns. It is crucial to broaden the scope of upcoming studies to encompass various racial groups and marginalized populations, taking into account other identities such as sexual orientation for a more comprehensive understanding. Moreover, researchers should incorporate additional variables like work experiences, workplace discrimination, and income to provide a more holistic perspective. Exploring different domains and dimensions of health outcomes, including mortality, should be a priority. Working conditions and work-life balance is another area that needs investigation [89–92]. Additionally, there is a pressing need to investigate specific mechanisms such as segregation, workplace discrimination, and the composition of the workplace that could elucidate differential effects. This research agenda holds the potential to shed light on the intricacies of Minorities’ Diminished Returns within the context of occupational classes.

## Conclusion

In summary, our study documented Minorities’ Diminished Returns in the relationship between professional occupational class and a wide range of health outcomes among middle-aged and older adults. Recognizing and addressing health disparities that are not explained by occupational differences but exist within similar occupational classes may reflect differential treatment of Non-Latino Black and Non-Latino White people within the job market. Continued research and policy efforts are warranted to foster a society where the benefits of similar occupations extend uniformly to all, irrespective of race.

## Data Availability

HRS data are publicly available here: https://hrsdata.isr.umich.edu/data-products/rand.
